# Anthocyanin-Rich Extract from Red Chinese Cabbage Alleviates Vascular Inflammation in Endothelial Cells and Apo E^−/−^ Mice

**DOI:** 10.3390/ijms19030816

**Published:** 2018-03-12

**Authors:** Hee Kyoung Joo, Sunga Choi, Yu Ran Lee, Eun Ok Lee, Myoung Soo Park, Kyu Been Park, Cuk-Seong Kim, Yong Pyo Lim, Jong-Tae Park, Byeong Hwa Jeon

**Affiliations:** 1 Research Institute of Medical Sciences, Department of Physiology, School of Medicine, Chungnam National University, 266 Munhwa-ro, Jung-gu, Daejeon 35015, Korea; hkjoo79@cnu.ac.kr (H.K.J.); sachoi@cnu.ac.kr (S.C.); lyr0913@cnu.ac.kr (Y.R.L.); y21c486@naver.com (E.O.L.); cskim@cnu.ac.kr (C.-S.K.); 2 Preclinical Research Center, Chungnam National University Hospital, Daejeon 35015, Korea; nova38@hanmail.net; 3 Department of Food Science and Technology, Chungnam National University, Daejeon 34134, Korea; pkb460@cnu.ac.kr (K.B.P.); jtpark@cnu.ac.kr (J.-T.P.); 4 Department of Horticulture, Chungnam National University, Daejeon 34134, Korea; yplim@cnu.ac.kr

**Keywords:** anthocyanin-rich red Chinese cabbage, apolipoprotein E-deficient mice, in vivo imaging, vascular cellular adhesion molecule, vascular inflammation

## Abstract

Anthocyanins, the most prevalent flavonoids in red/purple fruits and vegetables, are known to improve immune responses and reduce chronic disease risks. In this study, the anti-inflammatory activities of an anthocyanin-rich extract from red Chinese cabbage (ArCC) were shown based on its inhibitory effects in cultured endothelial cells and hyperlipidemic apolipoprotein *E*-deficient mice. ArCC treatment suppressed monocyte adhesion to tumor necrosis factor-α-stimulated endothelial cells. This was validated by ArCC’s ability to downregulate the expression and transcription of endothelial adhesion molecules, determined by immunoblot and luciferase promoter assays, respectively. The regulation of adhesion molecules was accompanied by transcriptional inhibition of nuclear factor-κB, which restricted cytoplasmic localization as shown by immunocytochemistry. Administration of ArCC (150 or 300 mg/kg/day) inhibited aortic inflammation in hyperlipidemic apolipoprotein E-deficient mice, as shown by in vivo imaging. Immunohistochemistry and plasma analysis showed that the aortas from these mice exhibited markedly lower leukocyte infiltration, reduced plaque formation, and lower concentrations of blood inflammatory cytokines than those observed in the control mice. The results suggest that the consumption of anthocyanin-rich red Chinese cabbage is closely correlated with lowering the risk of vascular inflammatory diseases.

## 1. Introduction

Coronary artery disease (CAD) is common in aging populations. It is a leading cause of mortality worldwide and is predicted to remain a concern for many years [1]. CAD has been described as an epidemic, accounting for approximately 45% of all cardiovascular disease related deaths [2,3]. Chronic vascular diseases, such as atherosclerosis, result from complex, multistep immune reactions, which eventually lead to the formation of atheromatous plaques, vascular endothelial dysfunction, and disruption of blood flow [4,5]. Therefore, targeting inflammatory pathways in the vascular system to reduce CAD risk could be a decisive, preventative strategy.

Dietary factors that can regulate inflammation in the vascular system have been identified. Examples include flavonoids (isorhamnetin, kaempferol, and quercetin glycosides) and phenylpropanoid derivatives from turnip [6,7] and pak choi [8]; isothiocyanates (phenethyl- and benzyl-isothiocyanate) from Chinese cabbage, radishes, and watercress; sulforaphane from broccoli [9,10]; and indole alkaloids and sterol glucosides from broccoli and cauliflower [11]. These phytochemicals can inhibit enzymes involved in prostaglandin and leukotriene synthesis, prevent free radical formation, decrease proinflammatory cytokine production, and inhibit signaling pathways [12,13]. More recently, colored phytochemicals have garnered interest because they exhibit remarkable anti-inflammatory activities. Glucosinolates and carotenoids, found in green and orange plants, respectively, modulate the DNA binding of nuclear factor-κB (NF-κB), which mediates immune responses [14,15]. Anthocyanins are phenolic compounds found in red-orange- to blue-violet-colored plants. In blueberries, environmental stress factors upregulate synthases, leading to the accumulation of protective anthocyanins [16]. Blueberry extracts inhibite monocyte chemotactic protein-1 (MCP-1) expression in endothelioma cells by transcriptional regulation of MCP-1 and NF-κB, causing hemangioma reduction [17]. Anthocyanin-enriched fractions from strawberries induce an improvement of the lipid profile showing antioxidant capacity in human hepatoma G2 cells [18]. Furthermore, anti-diabetic effects of berry polyphenols indicate that its biological activities spread to a regulation of cellular metabolism beyond their mere antioxidant capacity in vivo [19].

Our preliminary data showed that reddish plant extracts regulated the level of reactive oxygen species without inducing cytotoxicity. These observations led us to postulate that anthocyanin-rich extract from red Chinese cabbage (ArCC) could modulate inflammation in the vascular system. In the present study, the effects of ArCC on vascular inflammation, monocyte adhesion, and expression of the adhesion molecules vascular cell adhesion molecule-1 (VCAM-1) and intercellular adhesion molecule-1 (ICAM-1) were evaluated in vitro. The anti-atherosclerotic effects of daily oral administration of ArCC in apolipoprotein E (Apo E)-deficient mice were also evaluated. The results of these studies provide important information on the role of anthocyanins, specifically red Chinese cabbage phytochemicals, as preventive agents against the chronic inflammatory disease, CAD.

## 2. Results

### 2.1. The Contents of the Anthocyanin-Rich Extract from Red Chinese Cabbage

**Figure 1:**
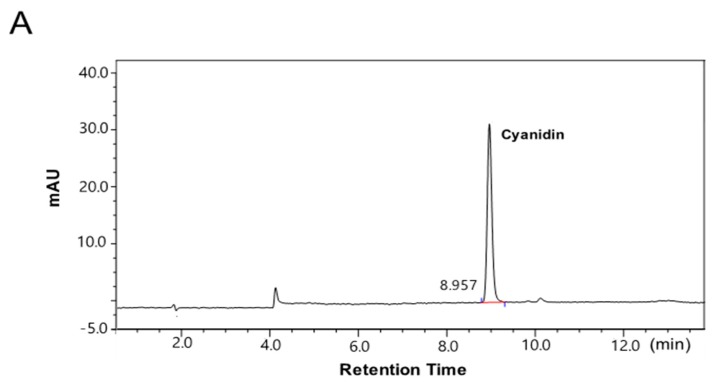

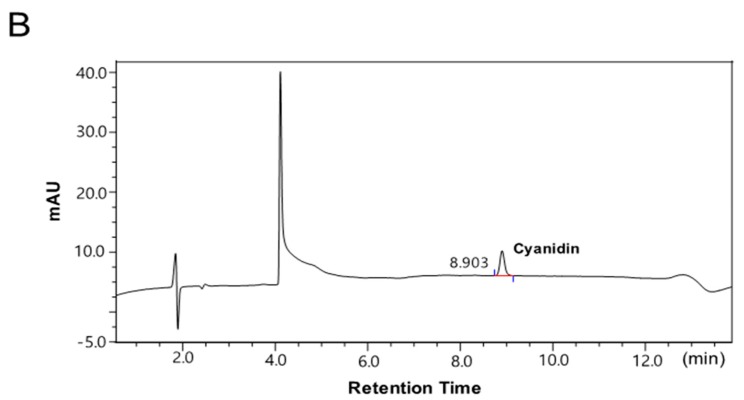
High-performance liquid chromatography (HPLC) profile of cyanidin from anthocyanin-rich extract from red Chinese cabbage (ArCC). HPLC chromatograms of a standard solution of 100 mg/L cyanidin (**A**) and cyanidin from ArCC (**B**) were recorded at 520 nm. mAU: milli Absorbance Unit.

Solid-phase chromatography using an adsorbent (Octadecyl-silica-A, 12 nm, 150 μm, YMC^®^, Kyoto, Japan) was used to purify the anthocyanin-rich extract from red Chinese cabbage. As determined by high-performance liquid chromatography (HPLC), the main anthocyanin of the extract was cyanidin, at 186 mg cyanidin/g dried powder (Figure 1). The other anthocyanins, pelargonidin and malvidin, were not detected in the ArCC.

### 2.2. ArCC Treatment Suppresses VCAM-1 Expression and Its Transcriptional Regulation in Tumor Necrosis Factor-α (TNF-α)-Stimulated Human Umbilical Vascular Endothelial Cells (HUVECs)

The effects of ArCC on cultured human umbilical vascular endothelial cells (HUVECs) were initially tested. The pH of the culture medium was unchanged after addition of ArCC at 10–1000 μg/mL, remaining at a ~7.4. ArCC treatment also preserved HUVEC viability, with >95% of cells surviving for 72 h at concentrations up to 1000 μg/mL. The analysis of real-time viability was used to determine ArCC concentrations in subsequent in vitro and in vivo studies (supplementary Figure S1).

**Figure 2:**
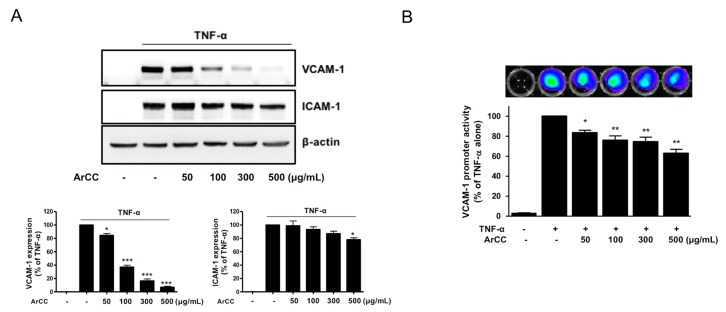
ArCC treatment suppresses adhesion molecule expression and transcriptional activation of vascular cell adhesion molecule-1 (VCAM-1) in TNF-α-stimulated human umbilical vein endothelial cells (HUVEC). (**A**) Immunoblotting for VCAM-1 and intercellular adhesion molecule-1 (ICAM-1) using lysates from tumor necrosis factor(TNF)-α-stimulated HUVECs treated with ArCC at the indicated concentrations. β-actin was used as the loading control. Relative changes in VCAM-1 (left) and ICAM-1 (right) expression levels were calculated based on densitometric scanning of each band. (**B**) Effects of ArCC on transcription of *VCAM-1* were measured via luciferase reporter assay. Human embryonic kidney (HEK) 293T/VCAM-1-luc cells (4 × 10^4^ cells/well) were plated in a black, 96-well plate, followed by treatment with various concentrations (0 to 500 μg/mL) of ArCC, followed by stimulation with TNF-α for 6 h. The bioluminescent images were acquired following the addition of 150 μg/mL d-luciferin substrate (upper). The luciferase activity, in units of total flux (p/s = photons/second) was quantified using an IVIS^®^ Lumina XRMS (X-ray multi-species optical imaging system) and analyzed by Living Image Software 4.4 (PerkinElmer) (lower). Values are expressed as mean ± SEM (*n* = 3). * *p* < 0.05, ** *p* < 0.01, *** *p* < 0.001, compared with TNF-α-only, as calculated using one-way analysis of variance followed by Bonferroni’s multiple comparison tests.

To address whether the ArCC treatment regulates the expression of adhesion molecules in TNF-α-stimulated HUVECs, expression levels of both VCAM-1 and ICAM-1 were analyzed. As shown in Figure 2A, following treatment with ArCC, statistically significant reductions occurred in a dose-dependent manner. An 83% suppression of VCAM-1 expression was observed with 300 μg/mL ArCC compared to expression with TNF-α treatment alone. Expression of the other adhesion molecule, ICAM-1, decreased with increasing ArCC concentration, although the effect was not as large, requiring 500 μg/mL ArCC for a 20% reduction. We were intrigued by the observations that ArCC treatment caused a dramatic inhibition of VCAM-1 in TNF-α-stimulated HUVECs (Figure 2A), which raised the possibility that ArCC treatment might decrease transcription of *VCAM*-*1.* We explored this possibility by determining the effect of ArCC treatment on *VCAM*-*1* promoter-luciferase reporters, and the results are shown in Figure 2B. Indeed, a significant decrease in the transcriptional activity of the *VCAM*-*1* promoter in a dose-dependent manner was observed; the level of luciferase decreased by 27% at 300 μg/mL ArCC (Figure 2B). Representative luminescence images also support the down-regulation of *VCAM-1* transcription after treatment with ArCC. These results indicate that the ArCC-mediated decrease in expression of adhesion molecules was caused, at least partially, by the regulation of transcription.

### 2.3. ArCC Treatment of TNF-α-Stimulated HUVECs Inhibited the Transcriptional Activity of NF-κB and Its Nuclear Localization

**Figure 3:**
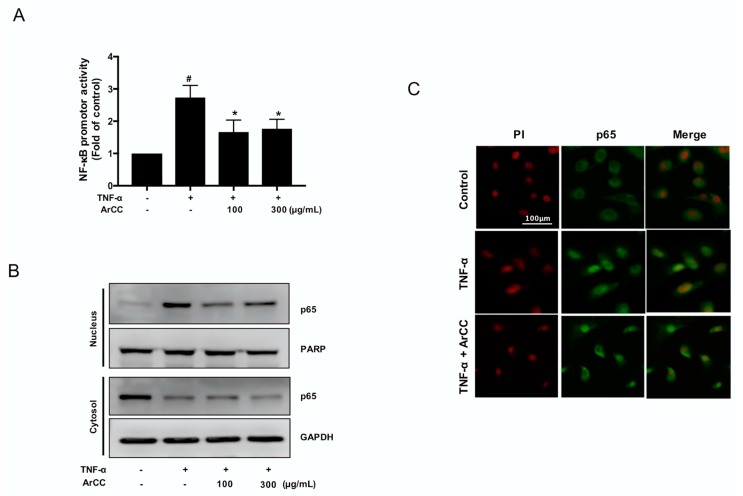
ArCC treatment significantly inhibited TNF-α-induced nuclear factor-κB (NF-κB) activity with translocation of NF-κB from the nucleus to the cytosol. (**A**) Transcriptional activity of NF-κB in TNF-α-stimulated HUVECs following treatment with 100 or 300 μg/mL of ArCC for 6 h as determined by luciferase reporter gene assay. Data are mean ± SE (*n* = 3). * Significantly different (*p* < 0.05) compared with TNF-α-only, ^#^ *p* < 0.05 compared with control by one-way ANOVA followed by Bonferroni’s test. (**B**) Immunoblotting for p65 NF-κB using nuclear fraction prepared from TNF-α-stimulated HUVECs following treatment with 100 or 300 μg/mL of ArCC for 6 h. The blot was stripped and re-probed with anti-poly (ADP-ribose) polymerase (PARP) antibody and anti-glyceraldehyde 3-phosphate dehydrogenase (GAPDH) to ensure equal protein loading without cross-contamination of the nuclear and cytoplasmic fractions. (**C**) Immunocytochemical analysis for p65 NF-κB localization in TNF-α-stimulated HUVECs following 6-h of treatment with 300 μg/mL of ArCC. Staining for p65 NF-κB and DNA is indicated by green and red fluorescence, respectively. p65 NF-κB localization was observed using a fluorescence microscope at 100× magnification

During vascular inflammation, the genes expression of adhesion molecules on endothelial cells is regulated by the NF-κB [20]. Next, the effect of ArCC treatment on NF-κB activity in TNF-α-stimulated HUVECs was determined using a luciferase reporter gene assay, and the results are shown in Figure 3A. A decrease in NF-κB transcriptional activity was observed in ArCC-treated HUVECs. Similarly to the expression of adhesion molecules (Figure 2A), ArCC caused a downregulation of NF-κB transcriptional activity. The TNF-α-induced initial activation was followed by a 40.7% inhibition at the 6-h time point (Figure 3A). To confirm inhibition of NF-κB in TNF-α-stimulated HUVECs under this experimental condition, the effect of ArCC treatment on nuclear translocation of p65 NF-κB was determined by immunoblotting for p65 NF-κB using nuclear fractions prepared from TNF-α-stimulated HUVECs treated with ArCC for different time periods. As shown in Figure 3B, treatment with 100 μg/mL of ArCC caused a marked decrease in the nuclear level of p65 NF-κB, which was evident at the 6-h time point. Immunocytochemistry to further examine the effect of ArCC treatment on the nuclear/cytoplasmic localization of p65 NF-κB was performed. As shown in Figure 3C, in TNF-α-stimulated HUVECs, p65 NF-κB immunostaining was evident in both nucleus and cytoplasm. The p65 NF-κB staining in a large fraction of ArCC-treated HUVECs was restricted to the cytoplasm, indicating inhibition of its nuclear translocation (Figure 3C). These results indicate that ArCC inhibited nuclear translocation of NF-κB in TNF-α-stimulated HUVECs.

### 2.4. ArCC Treatment Inhibits Monocyte Adhesion on TNF-α-Stimulated HUVECs

**Figure 4:**
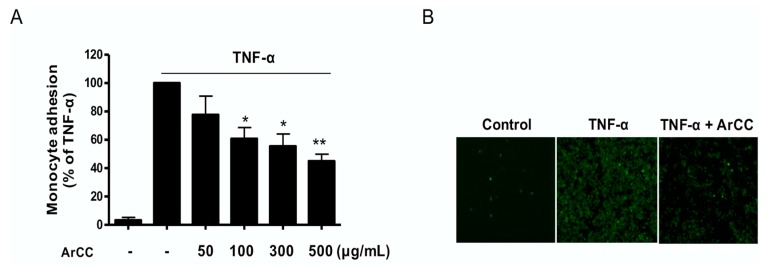
ArCC treatment inhibits monocyte adhesion on TNF-α-stimulated HUVECs. HUVECs were pretreated with ArCC for 1 h, followed by treatment with TNF-α for 18 h. Fluorescent probe-labeled U937 monocytes were added onto the TNF-α-stimulated HUVECs and then incubated for 2 h. (**A**) The number of U937 cells adhering to the HUVECs was determined using a fluorometer. (**B**) Adherent U937 cells observed using a fluorescence microscope at 200× magnification. Data are representative of three independent experiments. Results are presented as means ± SEM (*n* = 3). * *p* < 0.05, ** *p* < 0.01 compared with TNF-α-treated group as determined by one-way analysis of variance followed by Bonferroni’s multiple comparison tests.

Due to its remarkable regulation of adhesion molecule expression, we next determined the functional effect of ArCC on monocyte adhesion. The results revealed that adhesion of fluorescent-labeled monocytes to activated HUVECs was significantly inhibited in a dose-dependent manner; pretreatment with 300 μg/mL ArCC decreased fluorescent signal by 46% compared to that with TNF-α alone. (*p* < 0.05 and 0.01; Figure 4A), corroborating the VCAM-1 expression data. These results were confirmed under fluorescent microscopy, as shown in Figure 4B. These results indicate that ArCC is a potential inhibitor of adherence of circulating monocytes to damaged endothelial cell surfaces and a potential regulator of vascular inflammation.

### 2.5. Oral Administration of ArCC to Hyperlipidemic Apolipoprotein E-Deficient (ApoE^−/−^) Mice

Next, we designed animal experiments to observe whether (a) ArCC administration affects lipid profiles of western-type, atherogenic diet (WD) ApoE^−/−^ mice, (b) ArCC administration affects vascular inflammation, including production of circulating inflammatory factors in blood, and adhesion molecules on the surface of the aorta, and (c) ArCC administration ultimately inhibit plaque formation in the aortas of ApoE^−/−^ mice. To address these questions, we administered ArCC daily by gavage to ApoE^−/−^ mice. The ArCC dose was selected according to the weight of compound that corresponds to 30 mg/kg cyanidin-3-*O*-glucoside (C3G), which inhibits mastitis and improves neurotoxicity in mice [21,22]. Considering that our ArCC contains 18% cyanidin, 167 mg/kg ArCC was calculated as the equivalent dose. A treatment schedule of ArCC (i.e., low, 150 mg/kg or high, 300 mg/kg) was used to demonstrate the suppression of inflammation in vivo.

### 2.6. ArCC Administration Regulates Lipid Profile Changes in Blood of ApoE^−/−^ Mice

The body weights and blood pressure of the control and experimental groups of ApoE^−/−^ mice were recorded periodically to observe the physiological changes caused by ArCC treatment. As listed in Table 1, the average body weights and blood pressures of the control and WD mice showed significant differences throughout the experimental period (body weight, 30.7 ± 0.7 g vs. 34.3 ± 0.9 g, blood pressure, 108.3 ± 4.9/77.1 ± 9.7 vs. 134 ± 3.6/88.2 ± 7.2 mmHg, # *p* < 0.05) without any other physiological differences. A significant reduction in body weight and blood pressure was observed in ApoE^−/−^ mice when treated with high-dose ArCC (body weight, 32.0 ± 0.5 g, blood pressure, 103.5 ± 8.8/71.3 ± 0.5 mmHg, * *p* < 0.05), with values being comparable to those of the statin-treated group (body weight, 31.1 ± 0.3 g, blood pressure, 106.2 ± 7.4/85.6 ± 7.3 mmHg, * *p* < 0.05). As shown in Table 2, WD ApoE^−/−^ mice had significantly elevated plasma total cholesterol (TC) and low-density lipoprotein (LDL) + very low-density lipoprotein (VLDL) cholesterol concentration mice (TC, 527.5 ± 9.7 mg/dL; LDL + VLDL, 490.1 ± 5.1 mg/dL; # *p* < 0.05) compared to those concentrations in ApoE^−/−^ mice fed normal chow (TC, 361.8 ± 6.1 mg/dL LDL + VLDL, 316.9 ± 9.3 mg/dL). Treatment with ArCC remarkably lowered the lipid profiles of the WD ApoE^−/−^ mice. For instance, the levels of both LDL + VLDL and total cholesterol were decreased in the plasma of high-dose ArCC-fed mice (TC, 466.6 ± 9.7 mg/dL; LDL + VLDL, 438.6 ± 8.9 mg/dL; * *p* < 0.05), and these values were statistically different from those of hyperlipidemic ApoE^−/−^ mice.

### 2.7. ArCC Administration Inhibits Vascular Inflammation of WD-Fed ApoE^−/−^ Mice

**Figure 5:**
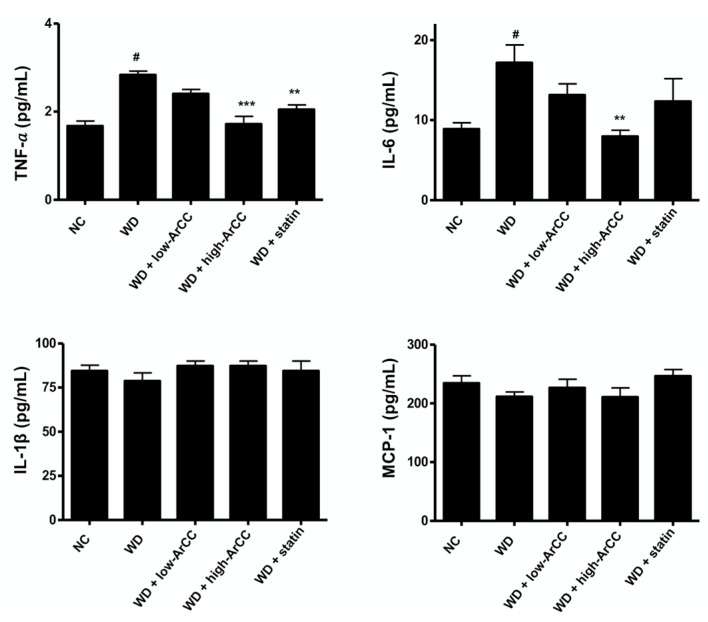
ArCC administration reduces inflammatory cytokine levels in plasma of western diet ApoE^−/−^ mice. The levels of plasma cytokines from ApoE^−/−^ mice at the indicated conditions were measured by multiplex. ApoE^−/−^ mice were fed a normal or western-type diet with or without ArCC for 12 weeks. Data are representative of experiments with at least eight mice in each group. ^#^ *p* <0.05 versus NC; ** *p* < 0.01, *** *p* < 0.001 versus WD as determined by one-way analysis of variance followed by Bonferroni’s multiple comparison tests.

Inflammatory cytokines have been implicated in the progress of vascular lesions and have a profound influence on the pathogenesis of atherosclerosis [23]. To test whether ArCC administration affected vascular inflammation, including the production of circulating inflammatory factors in the blood, inflammatory cytokine levels in the plasma of WD or ArCC-treated hyperlipidemic ApoE^−/−^ mice were analyzed by Luminex multiplex assay. As shown in Figure 5, high-dose ArCC treatment significantly decreased the levels of plasma cytokines compared with those in WD ApoE^−/−^ mice. For example, the level of TNF-α and interleukin (IL)-6 in high-ArCC-treated ApoE^−/−^ mice reached basal levels. In addition, the levels of other cytokines in high-ArCC-treated ApoE^−/−^ mice were comparable with those of the ApoE^−/−^ mice fed with atorvastatin, the positive control agent.

**Figure 6:**
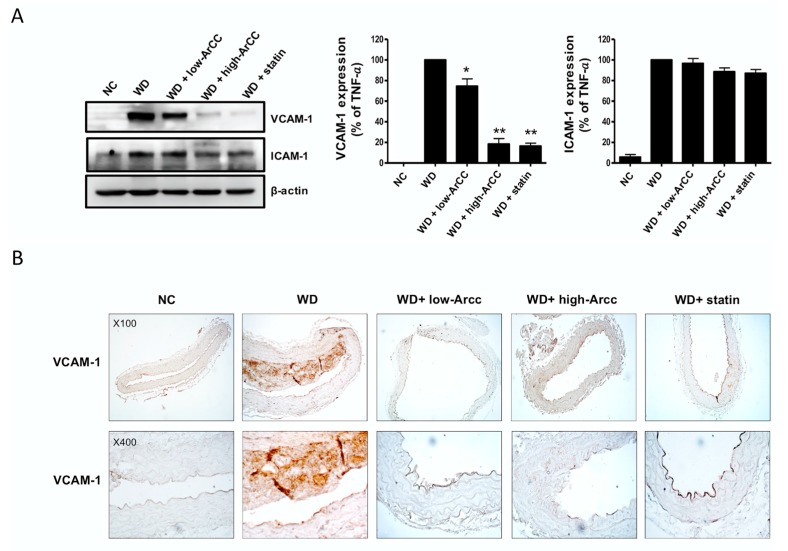
ArCC administration inhibits vascular inflammation of WD ApoE^−/−^ mice. (**A**) Immunoblotting for VCAM-1 and ICAM-1 using lysates from whole aortas of western diet (WD)-fed ApoE^−/−^ mice with ArCC treatment at the indicated conditions. β-actin was used as loading control. Relative changes in the expression levels were calculated based on densitometric scanning of each band. * *p* < 0.05, ** *p* < 0.01 versus WD as determined by one-way analysis of variance followed by Bonferroni’s multiple comparison tests. (**B**) Immunohistochemistry for VCAM-1 in the thoracic aorta of NC ApoE^−/−^ mice, WD ApoE^−/−^ mice, WD ApoE^−/−^ mice with ArCC administration (low or high), and WD ApoE^−/−^ mice with statin (*n* = 4). The aortas were sectioned and stained with anti-VCAM-1 antibody. Sectioned aortas were counter-stained with hematoxylin (Magnification, 100× or 400×).

The protein levels of adhesion molecules in the aortas of all groups of ApoE^−/−^ mice were examined by immunoblot, as shown in Figure 6A. The levels of the adhesion molecules VCAM-1 and ICAM-1 significantly increased in the aortas of WD ApoE^−/−^ mice compared to those in the aortas of normal chow (NC) ApoE^−/−^ mice. The expression levels of the adhesion molecules were remarkably suppressed by ArCC treatment. Densitometric analysis indicated that low-and high-ArCC treatment decreased VCAM-1 levels compared with those of WD ApoE^−/−^ mice (74.6 ± 8 and 18.45 ± 5, vs. 100.00, respectively, *p* < 0.05). Aortic ICAM-1 expression in high-ArCC-treated ApoE^−/−^ mice also decreased by 11.5%, although the difference did not reach statistical significance with low-ArCC treatment. VCAM-1 expression in WD ApoE^−/−^ mice was also observed, via immunohistochemical staining, in the blood vessel intima (atherosclerotic lesion), the endothelium covering the atherosclerotic lesion, and the endothelium outside the lesion (Figure 6B). Moreover, remarkable VCAM-1 expression was observed in smooth muscle cells within the tunica media under the lesions. Immunohistochemical staining patterns of VCAM-1 in ArCC-treated mice were also observed. A strong reduction was noted, especially inside the atherosclerotic lesion (Figure 6B), and, in mice treated with high-ArCC, even in the vessel media under the lesion. These results indicate that ArCC administration reduces aortic inflammation by inhibition of cytokine production and expression of adhesion molecules on the endothelial surface in atherogenic ApoE^−/−^ mice.

### 2.8. ArCC Administration Inhibits Plaque Formation in the Aortas of ApoE^−/−^ Mice

**Figure 7:**
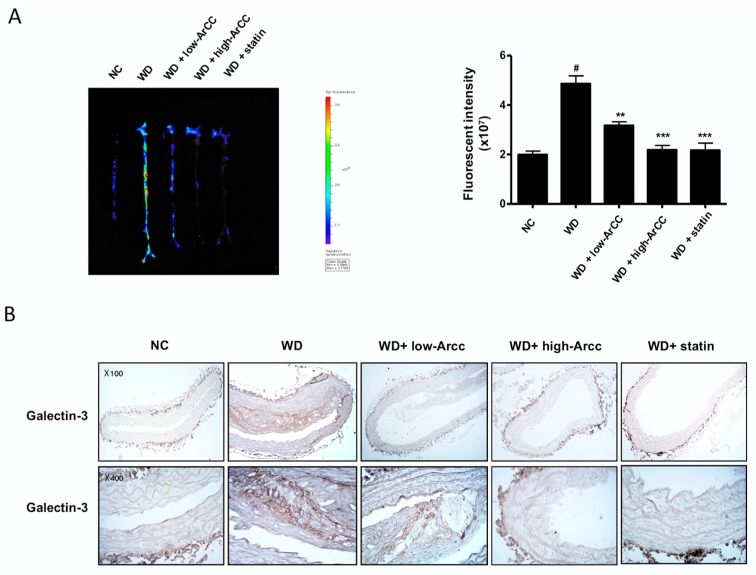
ArCC administration inhibits plaque formation in aortas of WD ApoE^−/−^ mice. (**A**) ApoE^−/−^ mice were injected with ProSense^®^750 EX via tail vein to visualize ex vivo aortic fluorescence imaging. Fluorescence imaging of aortas was quantified using an IVIS^®^ Lumina XRMS (X-ray multi-species optical imaging system) and analyzed by Living Image Software 4.4 (PerkinElmer). High signal intensities are shown in red/yellow/green/blue (Color scale). ^#^ *p* <0.05 versus NC; ** *p* < 0.01, *** *p* < 0.001 versus WD as determined by one-way analysis of variance followed by Bonferroni’s multiple comparison tests. (**B**) Immunohistochemistry for galectin-3 in the thoracic aorta of NC ApoE^−/−^ mice, WD ApoE^−/−^ mice, WD ApoE^−/−^ mice with ArCC administration (low or high), and WD ApoE^−/−^ mice with statin (*n* = 4). Aortas were sectioned and stained with anti-galectin antibody. Sectioned aortas were counter stained with hematoxylin (Magnification, 100× or 400×).

Because previous results have shown that ArCC inhibits vascular inflammation in vitro and in vivo, we confirmed the suppression of plaque formation in aortas from ArCC-treated ApoE^−/−^ mice. The images of the entire aortas from WD and ArCC-treated ApoE^−/−^ mice were acquired to quantify cathepsin-activatable fluorescence intensities, which indicate atherosclerotic lesions. As shown in Figure 7A, aortas from control ApoE^−/−^ mice exhibited strong fluorescence intensities over artery lesions, even in the iliac arteries. On the contrary, low-ArCC-treated mice showed decreased fluorescence intensities: a 60% decrease in thoracic, atherogenic lesions compared with those of WD aortas. Few fluorescent lesions were observed in the aortas of high-ArCC-treated ApoE^−/−^ mice, with weak signals remaining in the thoracic aortas. The suppression was comparable to that observed in the atorvastatin-treated ApoE^−/−^ mice. The results were in agreement with the fluorescent imaging, suggesting attenuated plaque formation by anti-inflammatory activity of ArCC in the aortas of ApoE^−/−^ mice. To identify macrophage infiltration in plaques, we analyzed the levels of galectin-3 in aortic tissue of ApoE^−/−^ mice. As shown in Figure 7B, galectin-3 expression increased in the aortas of WD ApoE^−/−^ mice compared with that in NC ApoE^−/−^ mice. On the contrary, galectin-3 expression was remarkably suppressed in the high-ArCC-treated group. Collectively, these results indicate that ArCC-mediated suppression of vascular inflammation in vivo was associated with down-regulation of inflammatory cytokines and adhesion molecules and reduced plaque formation in aortas of ApoE^−/−^ mice.

## 3. Discussion

In the present study, we demonstrated that ArCC prevents atherosclerotic vascular inflammation by regulating inflammatory factors in the blood. The ArCC-mediated anti-inflammatory effects were not restricted to cultured endothelial cells because the inhibition of plaque formation in the arteries of hyperlipidemic ApoE^−/−^ mice by daily ArCC administration correlated with a decrease in inflammatory cytokines and adhesion molecule expression on the surface of the arterial endothelium. Moreover, we showed for the first time that the aortas from ArCC-treated mice clearly exhibited decreased fluorescence intensities in the atherosclerotic lesions by in vivo imaging, supporting previous speculations that natural extracts containing anthocyanin may function as effective anti-inflammatory agents.

Many epidemiological studies have demonstrated that consumption of cruciferous vegetables rich in anthocyanins is associated with lower incidences of human chronic vascular inflammatory diseases, including cardiovascular disease, atherosclerotic coronary heart disease, and oxidative stress-related disorders [24,25,26].

The major sources of anthocyanins are naturally red and dark purple colored plants, such as berries, grapes, rice, and corn [27]. The dark pigment, cyanidin, which is the major compound in ArCC, is recognized as the most pharmaceutically effective anthocyanin. During digestion, the cyanidin is metabolized mainly to a glucuronide derivative, reaching peak plasma concentrations between 1 and 3 h. Approximately 80% of the cyanidin content in the intestines is in the stable form as C3G [28]. Suppression of inflammation-related cytokines and adhesion molecules [21,29] and induction of anti-oxidant activity by anthocyanin (mainly C3G) in cultured cells have been observed at 20–40 μmol/L [30]. Regulation of the blood lipids profile and inhibition of foam cell formation in the aorta have been shown after intraperitoneal administration of C3G at 10–40 mg/kg [21], and an improvement in plasma antioxidant capacity has been observed with oral administration at 1–2 g/kg diet (20–40 mg/day) [31]. After a 500 mg oral bolus of C3G, a peak serum concentration of 141 nM was observed after 1–2 h, with immediate absorption within 0.5–2 h in the stomach/intestine and an elimination half-life of 0.4 h, which explains the rapid delivery of C3G into the blood [32,33]. Therefore, the oral dosage of ArCC (i.e., 27 mg cyanidin/150 mg ArCC/kg, and 54 mg cyanidin/300 mg ArCC/kg) to ApoE^−/−^ mice in this study is reasonable to induce vascular anti-inflammation in vivo. The plasma concentrations of ArCC required for these anti-inflammatory effects and the reduction in atherosclerotic plaque formation may be achievable in humans. Based on body surface area conversion, 300 mg ArCC/kg in mice is equal to 12 mg/kg in humans, or the equivalent of 650 g of fresh red Chinese cabbage per day for a 60-kg person [34]. Although not reasonably achievable through consumption of Chinese cabbage, considering the traditional dietary habits of Koreans, which includes Kimchi with cabbage as a main ingredient, this concentration may be provided by a daily oral supplement.

The present study showed that the ArCC-mediated suppressed transcription and expression of adhesion molecules correlated with the remarkable decrease in monocyte adhesion to TNF-α-stimulated endothelial cells. The ArCC-mediated inhibition of inflammatory plaque formation in the aortas of hyperlipidemic ApoE^−/−^ mice paralleled the decrease in VCAM-1, although the effect on ICAM-1 was not as striking. The mechanism of the pharmacologic effects of ArCC on vascular adhesion molecules was linked to regulation of NF-κB [30]. The present study supports that nuclear translocation of p65 NF-κB was inhibited on treatment of TNF-α-stimulated HUVECs with ArCC. In addition, the ArCC-mediated inhibition of inflammation in the aorta was visualized with the broad inflammation probe, Prosense^®^ 750, instead of a specific probe against adhesion molecules. Although remarkable inhibition of vascular inflammation was caused by ArCC treatment, the anti-VCAM-1 positive sites in the atherosclerotic lesions did not exactly overlap the fluorescent-labeled loci. Therefore, the development of specific probes against each vascular inflammation-related adhesion molecules, such as VCAM-1, ICAM-1, and selectins, is required to systematically clarify the effects.

As preclinical efficacy testing of potential anti-inflammatory agents in vivo is an important step toward their use in humans, we determined the effect of ArCC on vascular inflammation in ApoE^−/−^ mice fed a hyperlipidemic diet. We found that oral administration of 150 or 300 mg ArCC/kg significantly reduced inflammation, as shown in the in vivo images. We also observed that the blood pressure and lipid profiles of ArCC-fed mice were remarkably reduced compared to those of WD ApoE^−/−^ mice, indicating that improvement in endothelium-dependent vasodilation was a possible mechanism of antihypertensive effects. In addition, the anti-atherogenic effects in ArCC-fed mice could be caused by direct anti-oxidant effects of ArCC that alter dietary lipid metabolism. The anti-oxidant activities of anthocyanin-rich foods, leading to the prevention of lipid peroxidation, have been well studied both in vitro and in vivo. Therefore, the potential blood pressure-lowering effect of anthocyanins in ArCC could also be an important mechanism contributing to the reduced risk of chronic vascular inflammation.

## 4. Materials and Methods

### 4.1. Materials

HUVECs and monocytic U937 cells were obtained from Clonetics (Walkersville, MD, USA), and HEK 293T epithelial cells were obtained from the American Type Culture Collection (#CCL-2, Rockville, MD, USA). Endothelial growth medium-2, Dulbecco’s modified Eagle’s medium, fetal bovine serum, and antibiotics were purchased from Gibco (Grand Island, NY, USA). Human tumor necrosis factor-α (TNF-α) was purchased from Sigma-Aldrich (St. Louis, MO, USA). Antibodies against cell adhesion molecules (i.e., VCAM-1 and ICAM-1) and β-actin were obtained from Santa Cruz Biotechnology (Dallas, TX, USA) and Sigma-Aldrich, respectively. An anti-mouse macrophage specific antibody (galectin-3, clone A3A12) was purchased from Abcam (Cambridge, UK). For analysis with the in vivo imaging system (IVIS), ProSense^®^ 750 FAST Fluorescent Imaging Agent was purchased from PerkinElmer (Santa Clara, CA, USA).

### 4.2. Preparation of ArCC

A commercial red Chinese cabbage cultivar (Kwonnong Carotene™, Kwonnong Seed Company, Chungju, Korea), cultivated in the autumn of 2015, was obtained from Kwonnong Seed Company and was stored at −20 °C until use. ArCC was prepared as described previously with some modification [35]. After thawing, the cabbage was blended and sonicated for 10 min (Qsonica, Melville, NY, USA) in 50% ethanol (*v/v*), and the soluble fraction was clarified by centrifugation (15,000× *g*) at 4 °C for 10 min. The soluble fraction was filtered through a hydrophilic polypropylene membrane, and then concentrated by rotary evaporation at 50 °C to remove ethanol. To prepare the anthocyanin containing extract, the fraction was chromatographed through a C18 column (YMC-DispoPack, YMC^®^, Kyoto, Japan). After washing the column with water, the adsorbed polar compounds, containing anthocyanins, were eluted with ethanol. The eluate was concentrated and lyophilized to obtain a powder. Anthocyanins were identified and quantified using the three standards, cyanidin, pelargonidin, and malvidin, according to the method described by Vasco et al. [36]. The amount of cyanidin calculated as the external standard equivalent was then multiplied by a molecular-weight correction factor to afford their specific quantities.

### 4.3. Monocyte-Endothelial Cell Adhesion Assay

The monocyte-endothelial cell adhesion assay was performed using suspension cultures of U937 cells, as described previously [37]. Briefly, confluent HUVECs (3 × 10^4^ cells) in 96-well plates were treated with various concentrations of ArCC, followed by stimulation with 15 ng/mL TNF-α for 18 h. The TNF-α-stimulated HUVECs were then incubated with fluorescent-labeled U937 monocytes for 2 h. The unbound U937 monocytes were removed by washing three times, and the fluorescence intensity of the bound cells was measured with a Glomax fluorometer (ex 485 nm; em 538 nm; Promega, Madison, WI, USA). Wells containing HUVECs alone were used as blanks. The bound U937 cells were also observed using a fluorescence microscope (Axiophot; Carl Zeiss, Oberkochen, Germany).

### 4.4. Promoter Assay

The effect of ArCC treatment on *VCAM-1* or *NF-κB* transcription was determined using luciferase reporter assay as described previously [37,38]. The HEK 293T/VCAM-1-luc cells were incubated with various concentrations of ArCC in an opaque-walled 96-well plate (4 × 10^4^ cells/well) for 1 h, followed by TNF-α stimulation for 6 h. The cells were treated with luciferin for 30 min at 37 °C, and the luminescence was measured by using a luminometer (Glomax, Promega, Madison, WI, USA). The resulting biophotonic reaction was also observed by using the IVIS^®^ Lumina XRMS imaging system and analyzed using Living Image Software 4.4 (PerkinElmer Inc., Waltham, MA, USA). Transiently transfected HUVECs with NF-κB-luciferase reporter plasmid and pCMV-pRL (Promega, Madison, WI, USA) internal control vector were treated with ArCC for 6 h, followed by stimulation with TNF-α. After the cells were harvested, the supernatant fraction was used to measure dual luciferase activity (Promega) with a luminometer.

### 4.5. Immunoblotting

Cells were treated with the specified concentrations of ArCC and lysed, as previously described [38]. The cell lysate was cleared by centrifugation at 12,000× *g* for 20 min at 4 °C, and the supernatant was used for immunoblotting. Proteins were resolved using sodium dodecyl sulfate-polyacrylamide gel electrophoresis (SDS-PAGE) and transferred onto a polyvinylidene fluoride membrane. Immunoblotting was performed using anti-VCAM-1 and anti-ICAM-1 antibodies (Santa Cruz Biotechnology, Dallas, Texas, USA), and a densitometric analysis was performed, normalizing to the β-actin signal. In some experiments, the cells were fractionated using a nuclear extraction kit from Pierce (Rockford, IL, USA) according to the manufacturer′s instructions. The supernatant fraction was collected and used for immunoblotting of p65 NF-κB. In addition, for immunoblot analysis of mouse tissue, the obtained aortas were homogenized in radioimmunoprecipitation assay (RIPA) buffer. The supernatants were cleared by centrifugation at 12,000× *g* for 20 min at 4 °C and were used for SDS-PAGE. 

### 4.6. Immunocytochemistry

HUVECs were cultured on coverslips, followed by treatment with 100 and 300 μg/mL of ArCC for 6 h and stimulation with TNF-α. After permeabilization with 0.1% Triton X-100, the cells were treated with anti-p65 NF-κB antibody (1:500 dilution; Santa Cruz Biotechnology, Dallas, TX, USA) for 2 h. Immunofluorescence staining was performed as described previously [38]. The cells were washed and incubated with Alexa Fluor 488-conjugated secondary antibody (1:1000 dilution; Molecular Probes, Eugene, OR, USA) for 1 h. After washing, the cells were counterstained with propidium iodide (PI) for 1 min. Finally, the cells were visualized under a fluorescence microscope (Axiophot; Carl Zeiss, Oberkochen, Germany).

### 4.7. Animal Experiments

ApoE^−/−^ mice, 14 weeks old, (ApoE^−/−^, C57BL/6J background; Jackson Laboratory, Bar Harbor, ME, USA) were used. The animals were kept at 24 °C with a 12-h day/night cycle with water and chow ad libitum. Mice were either fed a normal diet or a western-type diet (atherogenic diet) containing 21% fat, 34% sucrose, 19.5% casein, and 0.15% cholesterol (Envigo, Madison, MI, USA) for 12 weeks. The animal protocol was approved by the Ethics Committee of Animal Experimentation of Chungnam National University Hospital (CNUH-015-A0017, approved at 7 May 2015).

ApoE^−/−^ mice (*n* = 40, male) were randomly subdivided into five groups (*n* = 8 per group). The control group of animals was fed normal chow. The other groups (*n* = 32) were fed with WD. Of the WD mice, one group was provided 150 mg/kg/day ArCC (WD + Low-ArCC), one group with 300 mg/kg/day ArCC (WD + High-ArCC), and one group with 10 mg/kg/day atorvastatin as a positive control (WD + statin). Both ArCC and atorvastatin were fed by gavage.

### 4.8. Evaluation of Atherosclerotic Lesions

After 12 weeks, the animals were fasted for 24 h and then injected via tail vein with a fluorescent probe, ProsSense^®^ 750 (Perkin Elmer, Waltham, MA USA), which is activated by cathepsins in inflammatory cells. The Prosense^®^ 750 biomarker has been used previously to detect inflammation in animal models of atherosclerosis [39,40]. The mice were allowed to recover for 24 h. The mice were then anesthetized, and laparotomies were performed to obtain the thoracoabdominal aortas. Some of the aorta was trimmed and subjected to imaging analysis using the IVIS^®^ Lumina XRMS optical system (PerkinElmer, Waltham, MA, USA). Signal intensity was quantified as the photon flux (photons per second) within the region of interest by using Living Image software. Bright-field images were acquired to observe the aortic structure and determine the field of view. Then, ex vivo fluorescent imaging was performed to quantify fluorescent intensity of the Prosense^®^ 750 in the entire aorta, with excitation and emission wavelengths of 750 and 770 nm, respectively.

### 4.9. Plasma Analysis

Plasma samples were prepared and analyzed to determine total cholesterol and lipoprotein content (VLDL and LDL). For the determination of four inflammation-related cytokines (TNF-α, interleukin-1β, IL-6, and MCP-1) in plasma, MultiPlex kits (KOMA Biotech, Seoul, Korea) were used.

### 4.10. Histological Staining

Immunohistochemical staining for VCAM-1 and galectin-3 were performed in paraffin-embedded sections (3 μm) of aortic tissues. The sections were incubated with an anti-VCAM-1 (1:200) and anti-galectin-3 (1:200) antibody overnight at 4 °C. Horseradish peroxidase-conjugated goat-anti-rabbit and anti-mouse (1:1000) secondary antibodies were used. Specificity of the immunostaining was assessed by staining with nonimmune rabbit immunoglobulin G (IgG) and mouse IgG as negative controls. The sections were counterstained with hematoxylin, dehydrated, and mounted. Histological staining was analyzed with TS view 7 software (Microscope.com, Roanoke, VA, USA), and was digitalized using (Motic microscopy, Richmond, BC, Canada).

### 4.11. Statistical Analysis

Differences in the measured variables between the control and ArCC-treated groups were determined using a one-way analysis of variance, followed by Bonferroni’s test for multiple comparisons with the aid of GraphPad Prism software (version 5.0, GraphPad Software, La Jolla, CA, USA). A *p* value of <0.05 indicated statistical significance.

## 5. Conclusions

The results of the present study show that ArCC treatment regulates TNF-α-induced inflammatory markers in cultured endothelial cells and inhibits vascular inflammation in hyperlipidemic ApoE^−/−^ mice, without causing weight loss or other side effects. ArCC-mediated suppression of vascular inflammation in hyperlipidemic ApoE^−/−^ mice paralleled the inhibition of circulating inflammatory cytokines and plaque formation on the surface of the arterial endothelium and suppressed vascular adhesion molecule expression. This study also suggests that regular consumption of anthocyanin-rich red Chinese cabbage is useful in lowering the risk of chronic vascular inflammatory diseases, and highlights the need for improved cultivation of functional vegetables with anti-inflammatory activities.

## Figures and Tables

**Table 1:** Body weight and hemodynamic changes in ApoE^−/−^ mice after 12 weeks of ArCC administration.

Parameter	NC	WD	WD + Low-ArCC	WD + High-ArCC	WD + Statin
Start weight (g)	26.1 ± 0.6	25.2 ± 0.5	26.4 ± 0.6	26.2 ± 0.5	25.9 ± 0.4
Final weight (g)	30.7 ± 0.7	34.3 ± 0.9 ^#^	33.8 ± 0.6 ^#^	32.0 ± 0.5	31.1 ± 0.3
SBP (mmHg)	108.3 ± 4.9	134.1 ± 3.6 ^#^	106.5 ± 8.6 *	103.5 ± 8.8 *	106.2 ± 7.4 *
DBP (mmHg)	77.1 ± 9.7	88.2 ± 7.2	71.7 ± 6.8	71.3 ± 0.5	85.6 ± 7.3
HR (bpm)	320.8 ± 38.9	326.3 ± 26.2	331.3 ± 22.4	353.7 ± 42.6	325.1 ± 32.3

NC; normal chow, WD; western type diet, SBP; systolic blood pressure, DBP; diastolic blood pressure, HR; heart rate. All values represent mean ± SEM, *n* = 8 animals per group. ^#^ *p* < 0.05 versus NC, * *p* < 0.05 versus WD.

**Table 2:** Plasma lipids in ApoE^−/−^ mice after 12 weeks of ArCC administration.

Plasma Lipids	NC	WD	WD + Low-ArCC	WD + High-ArCC	WD + Statin
TC (mg/dL)	361.8 ± 6.1	527.5 ± 9.7 ^#^	510.5 ± 6.9	466.6 ± 9.7 *	395.9 ± 5.7 *
LDL + VLDL (mg/dL)	316.9 ± 9.3	490.1 ± 5.1 ^#^	477.7 ± 5.4 *	438.6 ± 8.9 *	364.7 ± 8.3 *

NC; normal chow, WD; western type diet, TC; total cholesterol, LDL + VLDL; low-density lipoprotein and very low-density lipoprotein. All values represent mean ± SEM, *n* = 8 animals per group. ^#^ *p* < 0.05 versus NC, * *p* < 0.05 versus WD.

## Supplementary Materials

Supplementary materials can be accessed at: http://www.mdpi.com/1422-0067/19/3/816/s1.

## Abbreviations

ArCC	anthocyanin-rich extract from red Chinese cabbage
ApoE^−/−^	hyperlipidemic apolipoprotein E-deficient
C3G	cyanidin-3-glucoside
ApoE	apolipoprotein E
HUVECs	human umbilical vein endothelial cells
VCAM-1	vascular cell adhesion molecule-1
ICAM-1	intercellular adhesion molecule-1
TNF-α	tumor necrosis factor-alpha
MCP-1	monocyte chemotactic protein-1
NF-κB	nuclear factor κB
CAD	coronary artery disease

## References

[1] Mathers C.D., Loncar D. (2006). Projections of global mortality and burden of disease from 2002 to 2030. PLoS Med..

[2] World Health Organization (2008). The Global Burden of Disease: 2004 Update.

[3] Beltrame J.F., Gaze D. (2012). Epidemiology of coronary artery disease. Coronary Artery Disease—Current Concepts in Epidemiology, Pathophysiology, Diagnostics and Treatment.

[4] Lichtman A.H., Binder C.J., Tsimikas S., Witztum J.L. (2013). Adaptive immunity in atherogenesis: New insights and therapeutic approaches. J. Clin. Investig..

[5] Ross R. (1999). Atherosclerosis—An inflammatory disease. N. Engl. J. Med..

[6] Romani A., Vignolini P., Isolani L., Ieri F., Heimler D. (2006). HPLC-DAD/MS characterization of flavonoids and hydroxycinnamic derivatives in turnip tops (*Brassica rapa* L. Subsp. sylvestris L.). J. Agric. Food Chem..

[7] Liang Y.S., Kim H.K., Lefeber A.W., Erkelens C., Choi Y.H., Verpoorte R. (2006). Identification of phenylpropanoids in methyl jasmonate treated Brassica rapa leaves using two-dimensional nuclear magnetic resonance spectroscopy. J. Chromatogr. A.

[8] Rochfort S.J., Imsic M., Jones R., Trenerry V.C., Tomkins B. (2006). Characterization of flavonol conjugates in immature leaves of pak choi [*Brassica rapa* L. Ssp *chinensis* L. (Hanelt.)] by HPLC-DAD and LC-MS/MS. J. Agric. Food Chem..

[9] Wu X., Zhou Q.H., Xu K. (2009). Are isothiocyanates potential anti-cancer drugs?. Acta Pharmacol. Sin..

[10] Hong E., Kim G.H. (2013). GC-MS Analysis of the Extracts from Korean Cabbage (*Brassica campestris* L. ssp. pekinensis) and Its Seed. Prev. Nutr. Food Sci..

[11] Schonhof I., Krumbein A., Bruckner B. (2004). Genotypic effects on glucosinolates and sensory properties of broccoli and cauliflower. Nahrung.

[12] Manach C., Scalbert A., Morand C., Remesy C., Jimenez L. (2004). Polyphenols: Food sources and bioavailability. Am. J. Clin. Nutr..

[13] Santangelo C., Vari R., Scazzocchio B., Di Benedetto R., Filesi C., Masella R. (2007). Polyphenols, intracellular signalling and inflammation. Ann. Ist. Super. Sanita.

[14] Fuentes F., Paredes-Gonzalez X., Kong A.T. (2015). Dietary Glucosinolates Sulforaphane, Phenethyl Isothiocyanate, Indole-3-Carbinol/3,3′-Diindolylmethane: Anti-Oxidative Stress/Inflammation, Nrf2, Epigenetics/Epigenomics and In Vivo Cancer Chemopreventive Efficacy. Curr. Pharmacol. Rep..

[15] Ciccone M.M., Cortese F., Gesualdo M., Carbonara S., Zito A., Ricci G., De Pascalis F., Scicchitano P., Riccioni G. (2013). Dietary Intake of Carotenoids and Their Antioxidant and Anti-Inflammatory Effects in Cardiovascular Care. Mediat. Inflamm..

[16] Routray W., Orsat V. (2011). Blueberries and Their Anthocyanins: Factors Affecting Biosynthesis and Properties. Compr. Rev. Food Sci. Food Saf..

[17] Atalay M., Gordillo G., Roy S., Rovin B., Bagchi D., Bagchi M., Sen C.K. (2003). Anti-angiogenic property of edible berry in a model of hemangioma. FEBS Lett..

[18] Forbes-Hernandez T.Y., Gasparrini M., Afrin S., Cianciosi D., Gonzalez-Paramas A.M., Santos-Buelga C., Mezzetti B., Quiles J.L., Battino M., Giampieri F. (2017). Strawberry (cv. *Romina*) Methanolic Extract and Anthocyanin-Enriched Fraction Improve Lipid Profile and Antioxidant Status in HepG2 Cells. Int. J. Mol. Sci..

[19] Edirisinghe I., Burton-Freeman B. (2016). Anti-diabetic actions of Berry polyphenols—Review on proposed mechanisms of action. J. Berry Res..

[20] Kempe S., Kestler H., Lasar A., Wirth T. (2005). NF-κB controls the global pro-inflammatory response in endothelial cells: Evidence for the regulation of a pro-atherogenic program. Nucleic Acids Res..

[21] Fu Y.H., Wei Z.K., Zhou E.S., Zhang N.S., Yang Z.T. (2014). Cyanidin-3-*O*-β-glucoside inhibits lipopolysaccharide-induced inflammatory response in mouse mastitis model. J. Lipid Res..

[22] Ke Z.J., Liu Y., Wang X., Fan Z.Q., Chen G., Xu M., Bower K.A., Frank J.A., Ou X.M., Shi X.L. (2011). Cyanidin-3-Glucoside Ameliorates Ethanol Neurotoxicity in the Developing Brain. J. Neurosci. Res..

[23] Ramji D.P., Davies T.S. (2015). Cytokines in atherosclerosis: Key players in all stages of disease and promising therapeutic targets. Cytokine Growth Factor Rev..

[24] Grassi D., Desideri G., Croce G., Tiberti S., Aggio A., Ferri C. (2009). Flavonoids, Vascular Function and Cardiovascular Protection. Curr. Pharm. Des..

[25] Garcia-Lafuente A., Guillamon E., Villares A., Rostagno M.A., Martinez J.A. (2009). Flavonoids as anti-inflammatory agents: Implications in cancer and cardiovascular disease. Inflamm. Res..

[26] Lockheart M.S., Steffen L.M., Rebnord H.M., Fimreite R.L., Ringstad J., Thelle D.S., Pedersen J.I., Jacobs D.R. (2007). Dietary patterns, food groups and myocardial infarction: A case-control study. Br. J. Nutr..

[27] Mazza G.J. (2007). Anthocyanins and heart health. Ann. Ist. Super. Sanita.

[28] Wallace T.C. (2011). Anthocyanins in Cardiovascular Disease. Adv. Nutr..

[29] Serra D., Paixao J., Nunes C., Dinis T.C., Almeida L.M. (2013). Cyanidin-3-glucoside suppresses cytokine-induced inflammatory response in human intestinal cells: Comparison with 5-aminosalicylic acid. PLoS ONE.

[30] Ferrari D., Speciale A., Cristani M., Fratantonio D., Molonia M.S., Ranaldi G., Saija A., Cimino F. (2016). Cyanidin-3-*O*-glucoside inhibits NF-κB signalling in intestinal epithelial cells exposed to TNF-α and exerts protective effects via Nrf2 pathway activation. Toxicol. Lett..

[31] Meskin M.S., Bidlack W.R., Davies A.J., Lewis D.S., Randolph R.K. (2003). Phytochemicals: Mechanisms of Action.

[32] De Ferrars R.M., Czank C., Zhang Q., Botting N.P., Kroon P.A., Cassidy A., Kay C.D. (2014). The pharmacokinetics of anthocyanins and their metabolites in humans. Br. J. Pharmacol..

[33] Passamonti S., Vrhovsek U., Vanzo A., Mattivi F. (2003). The stomach as a site for anthocyanins absorption from food. FEBS Lett..

[34] Reagan-Shaw S., Nihal M., Ahmad N. (2008). Dose translation from animal to human studies revisited. FASEB J..

[35] Joo H.K., Choi S., Lee Y.R., Lee E.O., Park M.S., Lim Y.P., Park J.T., Jeon B.H. (2017). Ethanol Extract of *Brassica rapa* ssp. pekinensis Suppresses Tumor Necrosis Factor-α-Induced Inflammatory Response in Human Umbilical Vein Endothelial Cells. J. Med. Food.

[36] Vasco C., Ruales J., Kamal-Eldin A. (2008). Total phenolic compounds and antioxidant capacities of major fruits from Ecuador. Food Chem..

[37] Lee K.M., Joo H.K., Lee Y.R., Park M.S., Kang G., Choi S., Lee K.H., Jeon B.H. (2016). *Ulmus davidiana* ethanol extract inhibits monocyte adhesion to tumor necrosis factor-α-stimulated endothelial cells. Integr. Med. Res..

[38] Lee Y.R., Kim K.M., Jeon B.H., Choi J.W., Choi S. (2012). The *n*-butanol fraction of *Naematoloma sublateritium* suppresses the inflammatory response through downregulation of NF-κB in human endothelial cells. Int. J. Mol. Med..

[39] Jaffer F.A., Libby P., Weissleder R. (2007). Molecular imaging of cardiovascular disease. Circulation.

[40] Chen J., Tung C.H., Mahmood U., Ntziachristos V., Gyurko R., Fishman M.C., Huang P.L., Weissleder R. (2002). In vivo imaging of proteolytic activity in atherosclerosis. Circulation.

