# Supplementary material for: Anthocyanin-Rich Extract from Red Chinese Cabbage Alleviates Vascular Inflammation in Endothelial Cells and Apo E−/− Mice
Source: Int J Mol Sci. 2018 Mar 12;19(3):816. doi: 10.3390/ijms19030816 (PMC5877677; doi:10.3390/ijms19030816)
Supplement: Supplementary file 1 [file ijms-19-00816-s001.pdf]

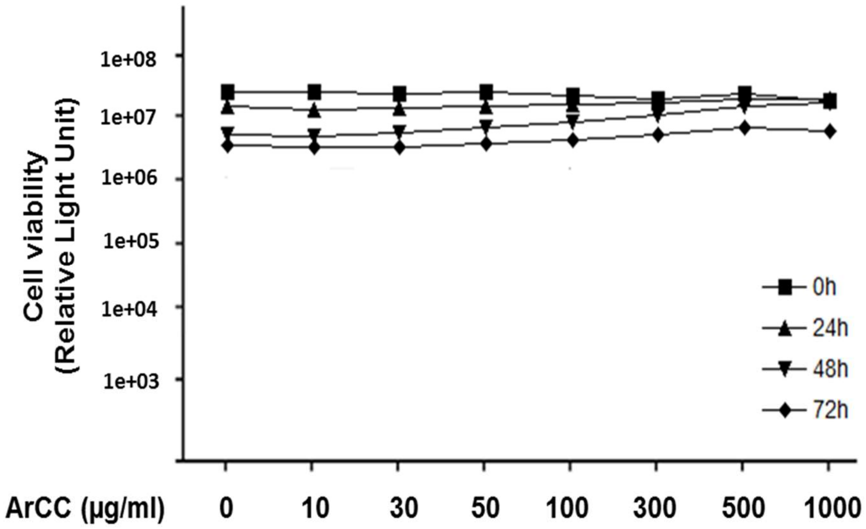

Figure S1. ArCC treatment did not cause reduction of viability at 10–1000 μg/mL in Stimulated Human Umbilical Vascular Endothelial Cells (HUVECs). The effect of the ArCC on HUVEC viability was determined using a bioluminescent assay and compared after 24, 48, and 72 h of treatment at the indicated concentrations in the presence of 15 ng/mL Tumor Necrosis Factor-α (TNF-α). Data are presented as means ± SEM (n = 3). Similar results were observed in replicate experiments.
